# A TinyML Soft-Sensor Approach for Low-Cost Detection and Monitoring of Vehicular Emissions

**DOI:** 10.3390/s22103838

**Published:** 2022-05-19

**Authors:** Pedro Andrade, Ivanovitch Silva, Marianne Silva, Thommas Flores, Jordão Cassiano, Daniel G. Costa

**Affiliations:** 1 Postgraduate Program in Electrical and Computer Engineering, Federal University of Rio Grande do Norte, Natal 59078-970, Rio Grande do Norte, Brazil; marianne.silva.086@ufrn.edu.br (M.S.); thommas.flores.101@ufrn.edu.br (T.F.); jordao.cassiano.009@ufrn.edu.br (J.C.); 2 Department of Computer Engineering and Automation, Federal University of Rio Grande do Norte, Natal 59078-970, Rio Grande do Norte, Brazil; 3 INEGI, Faculty of Engineering, University of Porto, 4200-465 Porto, Portugal; danielgcosta@fe.up.pt

**Keywords:** Internet of Things, Internet of Intelligent Vehicles, TinyML, soft sensor, air pollution, OBD-II

## Abstract

Vehicles are the major source of air pollution in modern cities, emitting excessive levels of CO_2_ and other noxious gases. Exploiting the OBD-II interface available on most vehicles, the continuous emission of such pollutants can be indirectly measured over time, although accuracy has been an important design issue when performing this task due the nature of the retrieved data. In this scenario, soft-sensor approaches can be adopted to process engine combustion data such as fuel injection and mass air flow, processing them to estimate pollution and transmitting the results for further analyses. Therefore, this article proposes a soft-sensor solution based on an embedded system designed to retrieve data from vehicles through their OBD-II interface, processing different inputs to provide estimated values of CO_2_ emissions over time. According to the type of data provided by the vehicle, two different algorithms are defined, and each follows a comprehensive mathematical formulation. Moreover, an unsupervised TinyML approach is also derived to remove outliers data when processing the computed data stream, improving the accuracy of the soft sensor as a whole while not requiring any interaction with cloud-based servers to operate. Initial results for an embedded implementation on the Freematics ONE+ board have shown the proposal’s feasibility with an acquisition frequency equal to 1Hz and emission granularity measure of gCO_2_/km.

## 1. Introduction

In most large cities around the world, air pollution has been regarded as a serious problem that has to be solved [1]. The World Health Organization (WHO) shows that 9.1 out of 10 people breathe air containing levels of pollutants superior to the WHO limits. It also shows that 4.2 million people die every year by cardiac and respiratory diseases caused by exposure to pollutants in the air. According to the UN, by the year 2030 the population living in urban areas will be 5 billion people, which leads us to consider the urgency of solutions to address this problem [2,3].

The cities represent only about 2% of the geographic area and accommodate over 50% of the world population; however, they are responsible for producing 80% of greenhouse gas emissions and consuming 80% of the world’s resources [4]. In this scenario, urban traffic is the major factor responsible for CO_2_ emissions because of its dependency on fossil fuels: Almost 23% of CO_2_ produced in Europe has its origin in the transport sector, while in the US, that equivalent percentage reaches 34% [5,6]. In this manner, the active monitoring of CO_2_ emissions is essential for controlling urban air quality. Nevertheless, such monitoring is often regarded as a complex issue because it involves some important challenges when measuring and processing gas emissions by vehicles. In this sense, the adoption of Internet of Things (IoT) solutions can be an important resource for such monitoring and analyses, since they can gather information that would not be available otherwise [7].

In recent years, soft-sensor approaches have been developed to address a lot of different problems by the proper sensing and processing of a group of data. With the IoT revolution and the availability of powerful hardware platforms at affordable prices [8,9], a new set of applications could be created by exploiting the combined processing of different types of data and computing new types of measurements that were not originally provided by the sensors. By conducting this, virtual sensor units could be created, creating an opening for many possibilities.

In order to monitor vehicular emissions and to perform related data analyses, which could be further exploited by any external system when assessing air quality in urban areas, this article proposes a soft-sensor approach to directly monitor vehicular emissions through an OBD-II (On-Board Diagnostics) interface and the Freematics ONE+ edge processing hardware [10]. Using an OBD-II scanner/reader, different types of data related to the vehicle’s engine will be retrieved from a vehicle, allowing real-time computations about vehicular emissions. Additionally, in order to provide higher accuracy when detecting noisy and outliers data, a TinyML (Tiny Machine Learning) algorithm will be proposed to process the computed data following an unsupervised learning paradigm that is oriented on data streams. The implemented TinyML algorithm has advantages over traditional statistical methods for detecting anomalies as there is no need for prior knowledge of the data and model training; that is, it is an unsupervised learning embedded on a resource-constrained device. Hence, since it is a soft-sensor approach, the created virtual sensor will continuously provide information about CO_2_ emissions, exploiting all proposed embedded algorithms.

Experimental results will be presented and discussed, providing indications about the effectiveness of the overall solution. Actually, when embedding the proposed low-cost approach into a hardware component to be attached onto cars, typically as a portable hardware unit to be provided by governments or environmental NGOs, this soft sensor allows a continuous and distributed monitoring of air pollution levels directly from pollution sources. Moreover, since an unsupervised learning paradigm is adopted for the defined TinyML, distinct from related works in this area, the development cost and time are reduced, eliminating dependencies to pruning approaches and, thus, fitting better for the scenario of monitoring of vehicular emissions. Putting all these together, the proposed approach may bring significant benefits in the long run.

The remainder of this article is organized as follows. Section 2 presents some important works related to this article, highlighting their main differences. Section 3 provides an overview about air pollution from vehicles, which is necessary when performing the required computations. Section 4 describes all elements of the proposed approach. Experimental results and discussions are presented in Section 5. Finally, conclusions and references are presented.

## 2. Related Works

Automobile factories have gradually increased the number of sensors and the capabilities of vehicle control systems in order to improve performance and increase safety for drivers [11,12]. The OBD-II (On Board Diagnostics) was launched as a system capable of self-diagnosing and alerting the driver about possible problems in the vehicle’s subsystems such as electronic components and the conditions of the emission of pollutants. Since 1996, all vehicles manufactured in the United States are required to support OBD-II, with other countries following the same trend [13]. With the implementation of the OBD-II system in major world markets, different approaches have been proposed to exploit the provided data to bring valuable information to the drivers.

With the increase in the volume of data generated by vehicles, the possibility of using IoT concepts aimed at vehicular applications has been largely promoted. In particular, IoT applications may use Machine Learning (ML) techniques to better process the available data, but usual ML solutions will stream raw or poorly processed data to a cloud server due to the complexities of employing ML algorithms locally. However, with the growth of data sources within an application, it becomes more difficult for a Cloud server to process the large volume of data generated, in addition to issues as latency and availability. Actually, cloud-only concentrated processing requires better transmission infrastructure, more power consumption, and greater system reliability. Therefore, it is reasonable to add “smart” functions to IoT devices so that they can process data and make decisions without passing all of the raw data to the cloud [14].

Basically, the principle of local processing can be perceived as a paradigm shift defined as “Edge computing”, with data processing being pushed to the edge of IoT devices and to the sensor data acquisition [15,16,17]. For such paradigms, although machine learning may be a constant, many applications may rely on resource-constrained devices such as microcontrollers, imposing some practical difficulties. Since the employed devices may have low processing power and restricted memory, more complex machine learning techniques may be prohibitive [18,19]. Therefore, the concept of TinyML arises as a method to fit ML models into resource-constrained devices [20,21,22], which tries to establish the best trade-off between the restricted hardware and a ML algorithm [23].

In parallel, in addition to the issues related to Machine Learning techniques implemented as embedded algorithms, with previous works addressing important challenges that arose from it, the literature concerning the soft sensor concept was also surveyed. Actually, with multi-sensors hardware units being affordably deployed with decent processing and memory capabilities, soft sensors have been created to provide new types of measurements in different types of applications. In [24], a warning system was developed to detect hazardous ground-level ozone concentrations, defining a soft-sensor approach that processes three meteorological factors to achieve a unified result, being supported by a neural network. For the work in [25], authors proposed a soft-sensor approach to process different water quality parameters, implementing machine learning to establish a correlation between input and output data. In [26], the soft sensor concept is exploited to avoid disasters in cities according to defined ontologies, transmitting alerts when necessary. Finally, some important performance issues for soft-sensor approaches were discussed in [27], particularly for smart cities scenario.

Since this article is focused on calculating CO_2_ emissions by cars, based on vehicle data, the literature was reviewed for works that comprised this keyword. Moreover, as TinyML is a key element of the proposed approach, it was also inserted into the search queries. However, since the TinyML paradigm is a novelty for the automotive sector, with few solutions in this area, the Machine Learning principle was considered instead in a more generic way, providing us with important information for comparison purposes.

Table 1 summarizes previous works that employ Machine Learning for the monitoring of CO_2_ emissions, with a particular concern for soft-sensor solutions.

Concerning Table 1, the work in [28] developed a platform for vehicular monitoring of CO_2_ emissions, with a mobile application being used to calculate emissions from retrieved OBD-II data. In [29], a platform for OBD-II pollution monitoring was proposed based on the crownd-sensing paradigm. Distinct from these two works, the authors in [30] employed several machine learning techniques to predict CO_2_ emissions from the retrieved data, but Edge computing was not exploited. For the work in [31], Freematics ONE+ hardware was adopted as an OBD-II reader to calculate the emission of pollutants according to the driver’s behavior, however the neural network was not implemented on Freematics, in a different way from this article. Finally, the work in [32] developed a platform that collects data via OBD-II and forwards it to a smartphone equipped with a fuzzy inference system to classify the driver’s behavior and to alert about CO_2_ emissions and fuel consumption, following a different development line.

Although promising, the presented works did not exploit the possibilities of TinyML when computing CO_2_ emissions by vehicles, which we believe will bring significant results at a reduced cost. Those works did not process data on the edge, or they were restricted by offline processing or the utilization of a mobile application on a smartphone. Filling this development gap, the proposed approach in this article can bring significant results for monitoring air pollution by vehicles and even within smart cities scenarios, supporting a new development trend.

## 3. Fundamentals of Vehicular Emissions

Rapid urban growth in the last decades has induced important concerns about air quality, especially in large cities [33,34]. This is due to the fact that air pollution may result in respiratory and cardiovascular diseases [35,36,37], which has demanded new solutions to monitor and even control the emission of pollutants in the air. Therefore, the monitoring of air pollution is expected to have a positive impact on human health along the time, especially when combined with actions aimed at the improvement of the quality of life in urban areas [38].

The emission of pollutants by vehicles is related to the ratio of the air-fuel mixture in the engine. When there is more fuel than the ideal amount to consume, the mixture is denominated “rich”. In this condition, part of the fuel is not consumed and it is expelled by the exhaust in the form of hydrocarbons (HxCx). In fact, HxCx is toxic and forms visible, thick smoke in places with a high concentration of cars. Additionally, sulfuric oxide (SO_2_) is a gas emitted from the exhaust of vehicles and can be used as an indication of traffic congestion in a region when analyzing the concentration of SO_2_ in a considered area. Moreover, carbon monoxide (CO) is also produced with the rich mixture. Such colorless, odorless, and tasteless CO gas is dangerous to living beings, because when it is inhaled and absorbed, it enters the bloodstream and associates with hemoglobin, preventing oxygen from being transported through the body, causing asphyxiation [39]. Finally, there is the expulsion of soot caused by the incomplete combustion in the engine, producing hydrocarbon particles [40].

On the other hand, when the air/fuel mixture is “poor”, nitrogen oxides are formed (NOx). In this case, with the expulsion through the exhaust, there is an association with more oxygen, making it more polluting. Nitrogen oxides can cause damages to the respiratory system and they can contribute to the formation of acid rain.

Some measures have been taken by governments to reduce the emission of pollutants into the atmosphere. The Paris Agreement, signed during the United Nations Framework Convention on Climate Change (UNFCCC) in 2016, provides for a reduction in and adaptation of greenhouse gas emissions [41]. To meet environmental requirements, a catalytic converter is used by cars to transform polluting gases into non-polluting substances: (i) Hydrocarbons are transformed into carbon dioxide (CO_2_) and water (H_2_O); (ii) carbon monoxide is transformed into carbon dioxide; (iii) NOx is reduced in nitrogen and oxygen.

Although all these gases are dangerous at different levels, there has been a major worldwide concern with CO_2_, also referred as greenhouse gas. Actually, many studies already associates CO_2_ emissions to the elevation of the average temperature of the planet. Hence, considering the emergency produced by ongoing climate change, this article is focused on the detection and monitoring of CO_2_ emitted by vehicles, potentially providing an important contribution when facing this stringent global challenge.

### 3.1. Calculating CO_2_ Emissions

In order to monitor continuous CO_2_ emissions by vehicles, a soft-sensor approach is proposed in this article. Basically, this approach will aim to measure the mass of carbon dioxide expelled by the vehicle’s exhaust in a certain time interval. This measurement is made indirectly since it is not provided by the OBD-II protocol. For that, CO_2_ emissions have to be computed from other sensor variables provided by the vehicles, each one defined by a different PID (Parameter Identification).

The sensors used to measure CO_2_ emissions are expressed in Table 2 with the respective units. Some vehicles have only the PID Manifold Absolute Pressure (MAP), while others have only PID Mass Air Flow (MAF). There are still some vehicle models that have both sensors. Thus, in order to comply with these particularities, we defined two different methods: one for the MAP and the other for the MAF.

#### 3.1.1. CO_2_ Calculation Using MAP

The first method exploits the Manifold Absolute Pressure sensor, which is slightly more complex than the second approach. For that, the development of a suitable mathematical formula was based on the Equation of Perfect Gases enunciated by Emile Clapeyron in the 19th century, which is expressed in Equation (1):

(1) PV=nRT

where the following is the case:

- *P* represents the pressure in the combustion chamber obtained by the MAP sensor in kPa or psi;
- *V* is the volume of combustion chambers in the engine cylinders by cubic centimeters (cm${}^{3}$);
- *R* is the general gas constant proposed by Clapeyron equals 8.3146 (cm${}^{3}$× MPa)/ (K × mol);
- *T* is the gas temperature obtained by the IAT (*Intake Absolute Temperature*) sensor in K;
- Finally, *n* is the number of moles in the sample.

This equation can be also written as expressed in Equation (2).

(2) $$n=\frac{PV}{RT}$$

Then, the mass of air ($m_{air}$) can be computed by multiplying *n* by the molecular weight (molar mass—$M_{air}$) of the air, as described in Equations (3)–(5).

(3) $$m_{air}=n\times M_{air}$$

(4) $$\frac{m_{air}}{M_{air}}=\frac{PV}{RT}$$

(5) $$m_{air}=\frac{PV}{RT}\times M_{air}$$

Actually, Equation (5) is valid if the Volumetric Efficiency (VE) of the engine equals 100%. Volumetric efficiency is defined as the ratio of the density of the air–fuel mixture admitted into the cylinder at atmospheric pressure to the density of the same volume of air in the intake manifold. The value of VE can be computed by Equation (6):

(6) $$VE\left(\%\right)=\frac{V_{intake}}{V_{nominal}}\times 100$$

where $V_{intake}$ is the real volume of intake air supported by the cylinders, and $V_{nominal}$ is the volume of the vehicle engine. In this manner, we achieve Equation (7) for the mass of air.

(7) $$m_{air}\left[g\right]=\frac{P\left[\mathrm{kPa}\right]V\left[{\mathrm{cm}}^{3}\right]}{R\left[\frac{{\mathrm{cm}}^{3}\mathrm{MPa}}{\mathrm{Kmol}}\right]T\left[K\right]}\times M_{air}\left[\frac{g}{\mathrm{mol}}\right]\times VE$$

After VE correction, CO_2_ is computed for a time interval, denominated as *mass air flow*. Then, it is necessary to include in the formula the number of revolutions per minute of the engine acquired by the OBD-II reader. The considered reference engine is a four-stroke engine in which two of the four phases of the cycle allow air to enter the interior, which is a very common configuration. After that, dividing the number of revolutions by 2 and, subsequently, dividing it by 60, the result in seconds is expressed in Equations (8) and (9):

(8) $$m_{af}=\frac{PV}{1000\times RT}\times M_{air}\times VE\times \frac{RPM}{2\times 60}$$

which are rearranged as follows.

(9) $$m_{af}\left[\frac{g}{s}\right]=\frac{P\times V\times M_{air}\times VE\times RPM}{1000\times 120\times R\times T}$$

After obtaining the mass air flow value, the fuel intake into the combustion chamber can be calculated as defined in Equation (10):

(10) $$V_{fuel}\left[L/s\right]=\frac{m_{af}\left[\frac{g}{s}\right]}{AFR\times \rho_{gas}\left[\frac{g}{L}\right]}$$

where AFR is the air-fuel ratio, and ρ represents the fuel density (Table 3).

Finally, it is possible to compute CO_2_ emissions *per* second multiplying $V_{fuel}$ by the carbon mass generated after burning 1 L of the fuel ($CO_{2PL}$), which is expressed in Equation (11).

(11) $$CO_{2}\left[g/s\right]=V_{fuel}\left[L/s\right]\times CO_{2PL}\left[g/L\right]$$

Table 3 specifies the CO_2_ burned *per* liter of fuel ($CO_{2PL}$) for the main types of fuel (Gasoline, Diesel, and Ethanol), as well as the values for fuel density [42].

#### 3.1.2. CO_2_ Calculation Using MAF

Some of defined equations in the last subsection were intended to estimate the mass air flow into the engine, since such information was not provided when computing the CO_2_ emission based on the PID MAP. However, vehicles that have PID MAF already provide such information; thus, it does not need to be computed according to Equation (9).

As a result, the estimation of the CO_2_ emission by a vehicle that provides the PID MAF is a simpler task. Thus, when programming the proposed soft-sensor approach, if the considered vehicle has both sensors (MAP and MAF), the emission of CO_2_ is calculated directly by exploiting the MAF provided by the OBD-II reader, saving resources and computing time. Actually, according to [43], emissions being calculated through the PID MAP or the PID MAF present similar values; thus, they could be adopted interchangeably.

## 4. Proposed Approach

In order to perform the desired computations, the Edge computing paradigm was exploited when gathering and processing all required data. Doing so, computing, communication and control functions take place in physical proximity to the data source [44], reducing the response time for critical applications and its sensitivity to delays [45,46]. We believe that such a paradigm is very suitable for the intended computations, opening many possibilities when integrating the proposed approach to broader smart city macro-systems.

The proposed soft-sensor solution was designed to be fully implemented inside the target vehicle. For that, both the measurements collected from the ECU (Engine Control Unit) sensors and the associated computations are performed using the microcontroller embedded in an OBD-II scanner. In this manner, the employed hardware had to be small and powerful enough to support such tasks, but some current off-the-shelf hardware platforms already meet those requirements. Anyway, different hardware components could be adopted according to the available budget, as well as other complementary parallel functions could be associated to the same hardware for performance issues.

**Figure 1:**
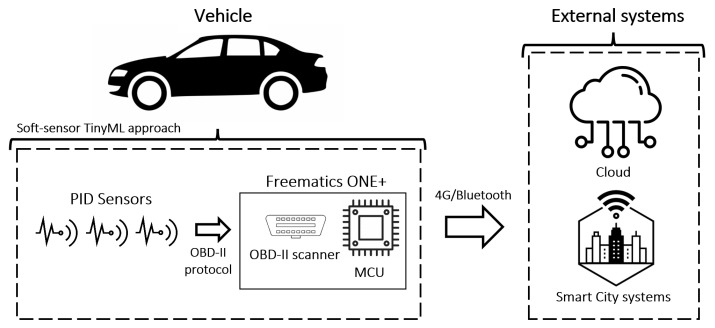
The conceptual architecture of the proposed approach.

Figure 1 depicts the conceptual architecture of the proposed soft-sensor solution. It is worth mentioning that although the CO_2_ calculation is performed following an Edge computing paradigm, the processed information can be transmitted in order to be further stored and processed by external systems (or even the Cloud), allowing different types of analyses when data are received from several vehicles adopting the proposed approach.

### 4.1. OBD-II Interactions with the Vehicles

Since all data to be processed comes from the vehicle through an OBD-II interface, a proper scanner hardware is a key element of the proposed approach. In fact, there are several commercial OBD-II scanners that can be used for vehicle diagnosis and data collection. In this article, we adopt the Freematics ONE Plus Model A (https://freematics.com/pages/products/freematics-one-plus/ (accessed on 13 May 2022)). Freematics is a vehicle telemetry platform that internally has an Espressif ESP32 (a fully programmable microcontroller) in addition to OBD-II connectivity, working in Operation Mode 1 of the protocol. Actually, this is a flexible but low-cost hardware decision that may support massive deployment of the proposed approach, but other more powerful hardware components could also be adopted according to the available budget.

The adopted Freematics ONE+ hardware allows storage on a microSD card or (internally) in a small Flash memory. Data transmissions can be performed in real time via 4G cellular networking or via Bluetooth, the latter being usually adopted along with a smartphone for Internet connectivity. Actually, Freematics ONE presents a higher commercial cost than most OBD-II scanners commonly found on the market, but it could be used without requiring a supporting platform such as Arduino or Raspberry Pi, reducing the overall cost of the implemented solution while reducing wiring and energy consumption.

The Freematics ONE+ is a hardware platform with an onboard storage capacity limit of a microSD card. In scenarios where network signal quality is a stringent variable or if there is no signal whatsoever, the device will continue to store information in its transmission buffer—a limited-capacity memory area. While this may be desirable in some situations, this may also cause some limitations if an application is attempting to transmit data that exceeds the device’s capacity. As far as performance discussion goes, it would be out of scope for this article to perform any network performance evaluations.

### 4.2. Data Acquisition and Initial Processing

**Figure 2:**
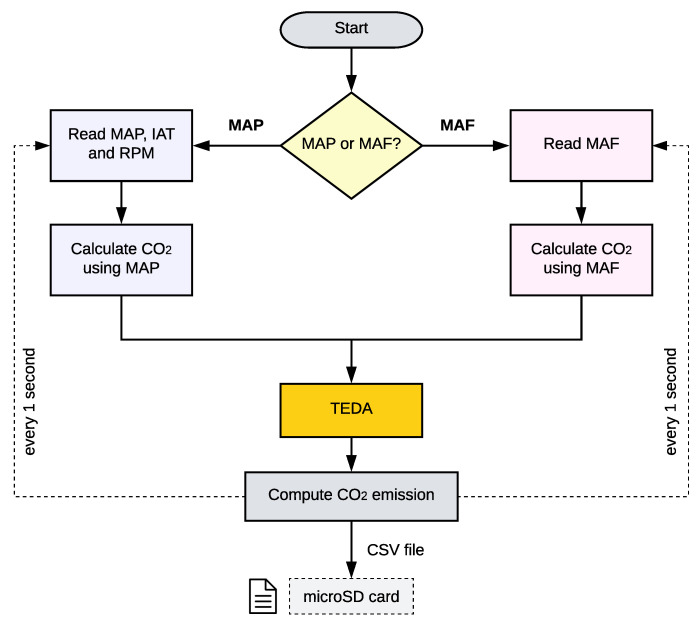
The processing flow of the proposed soft-sensor approach.

After the successful installation of the Freematics in the target vehicle, which has to be attached onto the vehicle’s OBD-II interface, a series of processing steps will take place, as depicted in the flowchart in Figure 2. Initially, the developed solution selects the PIDs of interest according to the target vehicle. Then, the selected PID is read every 1 s, which is the adopted reading frequency. As mentioned before, the list of available PIDs varies according to vehicles and manufacturers, with no standardization or fixed set of available PIDs.

Therefore, it has to be evaluated whether the vehicle has the MAP or the MAF PID (or even both), since it dictates the equations to be adopted for CO_2_ emission estimations. Then, according to the available PID, the sensors values are stored in the microSD memory card and the calculation of CO_2_ is performed following the corresponding proposed methods. The results after such processing is also stored in the microSD card, representing a pre-processing phase of the proposed approach. At this point, the computed results may have some accuracy issues that could compromise their relevance for air pollution monitoring, which led us to propose the TinyML step of the algorithm, referred as TEDA (Typicality and Eccentricity Data Analytics), as depicted in Figure 2.

The TEDA algorithm is intended to evaluate the presence of possible outliers in the time series. If the calculated sample is an outlier, it will be labeled as such for further analysis. Subsequently, the values of CO_2_ are used in the proposed TinyML method to predict future values. The great importance of having future values is the possibility of anticipating corrections for enrichment or depletion of the air fuel mixture in order to reduce the emission of pollutants when looking for an optimum point. All values directly read from the sensors and the values calculated by the defined algorithms were saved in the CSV format.

### 4.3. The TEDA Processing Step

In this article, we propose a Typicality and Eccentricity Data Analytics (TEDA)-based algorithm to improve the relevance of the achieved results. The idea is to implement an anomaly detection algorithm for data streams by exploiting typicality and eccentricity concepts. In short, typicality is the similarity of a sample to the rest of the set (based on distances between samples), while eccentricity is its opposite when indicating how different a sample is from the rest of the collected data [47]. In these definitions, a sample with high eccentricity indicates that it could be an outlier.

In order to measure the typicality and eccentricity of each new sample in the data stream, TEDA uses the sum of the geometric distances between the analyzed sample and the other samples in the set. The higher this value, the greater the eccentricity of the sample in relation to the others and, consequently, the lower the value of typicality. Moreover, this is employed when further processing CO_2_ emissions.

The modeling of the considered data stream can be given by an ordered vector $x=\left\{x_{1},x_{2},\cdots ,x_{k},\cdots \right\},∴x_{i}\;\epsilon \;R^{n},\;i\;\epsilon \;N$, where *k* represents the discrete time. The distance between $x_{i}$ and $x_{j}$ is given by $d(x_{i},x_{j})$, which can be the Euclidean distance, cosine distance, Mahalanobis distance, or any other reference. In this work, Euclidean distance is considered. Then, for the entire dataset up to the instant *k*, Equation (12) is defined, with $\pi_{k}\left(x\right)$ as the sum of the distances from a particular sample (*x*) to each of the other *k* elements.

(12) $$\begin{array}{c} \pi_{k}\left(x\right)=\sum_{i=1}^{k}d\left(x,x_{i}\right),\;k\geq 2 \end{array}$$

Therefore, the eccentricity ($\xi_{k}\left(x\right)$) of the data sample *x*, at the instant of time *k*, is defined by Equation (13).

(13) $$\begin{array}{c} \xi_{k}\left(x\right)=\frac{2\pi_{k}\left(x\right)}{\sum_{i=1}^{k}\pi_{k}\left(x_{i}\right)},\;k>2,\;\sum_{i=1}^{k}\pi_{k}\left(x_{i}\right)>0 \end{array}$$

This equation was rewritten in [47] so that the eccentricity could be calculated recursively, resulting in the definitions in Equation (14) for the eccentricity ($\xi_{k}\left(x\right)$) calculated at time *k* of the sample $x_{k}$ in relation to the other samples in the set. Additionally, $\mu_{k}$ is defined as the mean of *x* and ${\left[\sigma_{k}^{2}\right]}_{k}$ is the variance of *x* at time *k*.

(14) $$\begin{array}{c} \xi_{k}\left(x\right)=\frac{1}{k}+\frac{{\left(\mu_{k}-x_{k}\right)}^{T}\left(\mu_{k}-x_{k}\right)}{k\sigma_{k}^{2}} \end{array}$$

With Equation (14), the calculation is simplified to the distance of new input data $x_{k}$ and mean $\mu_{k}$. For that, $\mu_{k}\left(x\right)$ and $\sigma_{k}^{2}\left(x\right)$ values for each iteration are calculated recursively using the Equations (15) and (16).

(15) $$\begin{array}{c} \mu_{k}\left(x\right)=\frac{k-1}{k}\mu_{k-1}+\frac{1}{k}x_{k},\;\;\mu_{1}=x_{1} \end{array}$$

(16) $$\begin{array}{c} \sigma_{k}^{2}\left(x\right)=\frac{k-1}{k}\sigma_{k-1}^{2}+\frac{1}{k-1}{∥x_{k}-\mu_{k}∥}^{2},\;\;\sigma_{1}^{2}=0 \end{array}$$

Following the same reasoning, the typicality ($\tau_{k}\left(x\right)$) of the data sample *x*, at time *k*, is given by the eccentricity complement in Equation (17).

(17) $$\begin{array}{c} \tau_{k}\left(x\right)=1-\xi_{k}\left(x\right)=\frac{k-1}{k}-\frac{{\left(\mu_{k}-x_{k}\right)}^{T}\left(\mu_{k}-x_{k}\right)}{k\sigma_{k}^{2}} \end{array}$$

Finally, the normalized eccentricity and normalized typicality are given in Equations (18) and (19).

(18) $$\begin{array}{c} \zeta_{k}\left(x\right)=\frac{\xi_{k}\left(x\right)}{2},\;\sum_{i=1}^{k}\zeta_{i}\left(x\right)=1,\;k\geq 2 \end{array}$$

(19) $$\begin{array}{c} t_{k}\left(x\right)=\frac{\tau_{k}\left(x\right)}{k-2},\;\sum_{i=1}^{k}t_{i}\left(x\right)=1,\;k\geq 2 \end{array}$$

After defining the normalized eccentricity and normalized typicality values for each new input data, the next step is outlier detection. One of the simplest and most well-known methods in the literature is to use “mσ” as a threshold for classification. A sample will be considered an outlier if it is a certain amount *m* of standard deviations; however, it must be previously assumed that the data distribution is Gaussian.

In a dataset with a significant number of samples and for any data distribution, it is possible to use Chebyshev Inequality [48]. This inequality states that the probability of the samples being away from the mean is lower or equal to $1/m^{2}$. The work in [49] adapted it to use normalized eccentricity, as expressed in Equation (20).

(20) $$\zeta^{i}\left(x_{k}\right)\geq \frac{m^{2}+1}{2k}$$

Therefore, the value of *m* represents the threshold of sensitivity of the method. The larger the value of *m*, the less sensitive the algorithm will be. If the normalized typicality ($\zeta^{i}\left(x_{k}\right)$) is greater than the second term in Equation (20), being a true proposition, then $x_{k}$ will be an outlier. If typicality is lower, the proposition will be false, and the new data will not be an outlier.

The recursive feature of the TEDA algorithm provides an algorithm with low computational effort by using few memory and processing resources, because there is no previous processing or training of the data [50,51]. Furthermore, the iterative calculations of typicalities are based on simple arithmetic, with no computational complexity. Additionally, TEDA has others advantages over traditional statistical methods for detecting anomalies. There is no need for prior knowledge of the data (unsupervised learning); thus, it is widely used for data streams and time series, possessing the ability to detect changes in concept and evolution of concepts. To use the algorithm, it is not necessary to know the mathematical model or the data distribution, being an important advantage for some real-world problems [51].

At this point, considering the TEDA algorithm and all defined equations, the proposed approach could be fully implemented.

## 5. Evaluation Results

After the successful implementation of the proposed approach, an evaluation phase took place. The idea was to verify the practical operation of the created algorithms and their execution performance in real vehicles. Moreover, we wanted to extract the retrieved and processed data, verifying how it could be further exploited for air pollution analyses. The descriptions of such evaluation procedures are presented in next subsections.

### 5.1. Experimental Setup

In order to achieve valuable data to allow performance assessment of the proposed approach within the available research budget, the proposed algorithms were implemented in a single Freematics ONE+ hardware unit and tested in a vehicle provided by a volunteer. That vehicle was a Nissan Kicks model 2020, a modern popular vehicle that provides only the PID MAF (Mass Air Flow) sensor. During five consecutive days, from Monday to Friday, at the same time (06:30 a.m.), the vehicle moved freely in the city of Natal (Brazil), considering the same driver during this period and trips lasting less than 20 min. For this evaluation phase during rush hours in the selected city, both small streets and urban highways were taken, allowing variations in the performed speeds.

Considering the characteristics of the selected vehicle, with only the PID MAF available, the calculation of CO_2_ emissions expelled by the vehicle was based on Equation (10). Actually, the compatibility of the CO_2_ calculation using MAP or MAF has already been verified in a previous work by the authors of this article, as seen in [43], which reinforces the relevance of the achieved results in this section.

### 5.2. Retrieved Data

For the defined experimental setup, data could be retrieved from the vehicle to allow the calculation of CO_2_ emissions, according to the data definitions in Table 4. In fact, some data comes from the vehicle, while others are provided by Freematics (such as GPS Latitude and Longitude coordinates).

The obtained data through the implemented soft-sensor solution will be processed in different ways, with CO_2_ emissions being related to the Intake Air Temperature and the RPM as expressed in Equation (9). In particular, in this article, volumetric efficiency will be considered as a constant equals to 80%, a reasonable value previously discussed in [52]. In doing so, we achieve higher processing efficiency without significant loss of precision, but the volumetric efficiency could be also computed separately if required.

**Figure 3:**
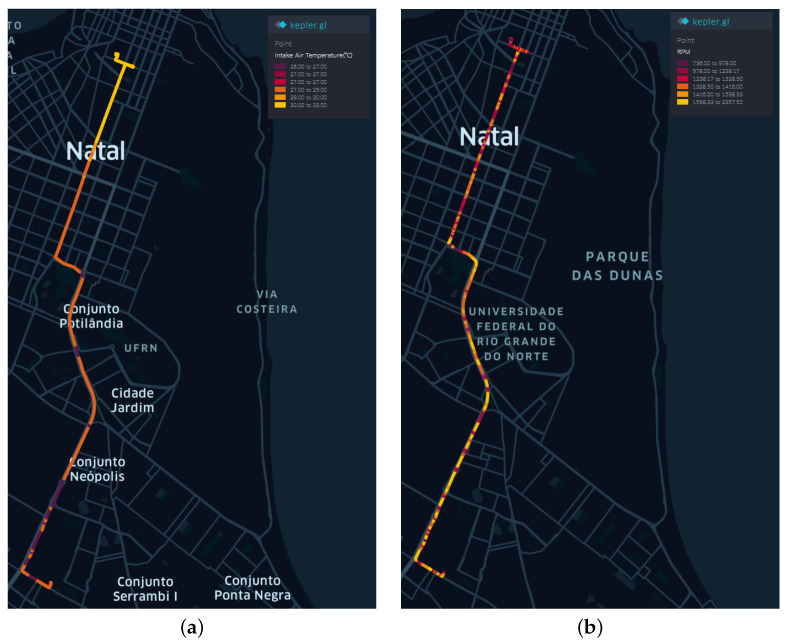
Vehicle variables on a day of the week—Tuesday. (**a**) Intake Air Temperature (°C) measurements. (**b**) RPM measurements.

The intake air temperature (°C) and the number of revolutions per minute (RPM) from the Tuesday dataset are shown in Figure 3 to illustrate the chosen path and variables.

Temperature data show that as time passes, the values increase, reaching a maximum level at the end of the trip, marked by the color yellow (Figure 3a). The revolutions per minute (RPM) data also shows a similar behavior, with an indication of the speed reductions in the higher speed sections, characterizing decelerations and gear changes (Figure 3b).

### 5.3. Evaluating CO_2_ Calculations

**Figure 4:**
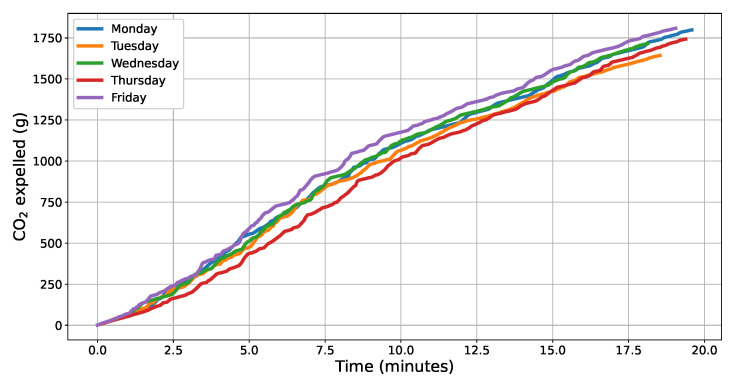
CO_2_ (g) expelled along the trip for each day of the week.

Considering the described experiment, the instantaneous values of CO_2_ calculated by the algorithm were cumulatively added to know the total mass of gas expelled by the vehicle along each route and on each day of the week. The curves of the cumulative sums for each of the 5 days of the experiment are shown in Figure 4. It is possible to observe that since the route, the vehicle, the driver, and the time of the day are the same, the patterns of the curves for each day of the week are somehow similar. Based on the displayed curves, Friday was the day with the highest total CO_2_ emission, reaching a value of 1808 g. The lowest CO_2_ mass value among the compared days was on Tuesday, reaching 1643 g.

**Figure 5:**
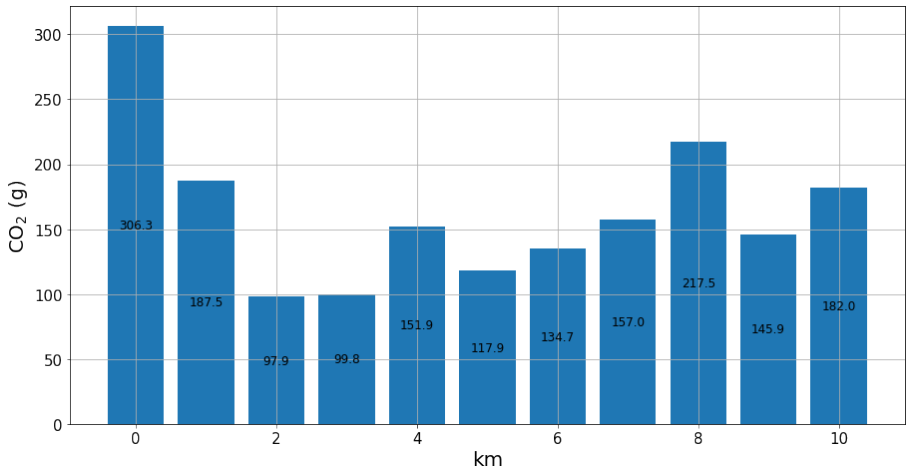
Mass of CO_2_ (g) expelled by the vehicle per kilometer driven.

Monday’s total CO_2_ emission was divided by kilometer as it is commonly represented in the literature, shown in Figure 5.

In fact, the developed soft-sensor approach presents values compatible with what is expected in the literature and in the automotive industry for the used vehicle in the experiment. According to [53], the average CO_2_ emission for cars manufactured between 2016 and 2020 varies between 100 g and 160 g per kilometer. The work in [54] indicates that a Nissan popular car emits an average of 232 g of CO_2_/km depending on maintenance conditions, type of road and driver behavior. Therefore, gCO_2_/km values computed by the soft-sensor approach are compatible with both references.

**Figure 6:**
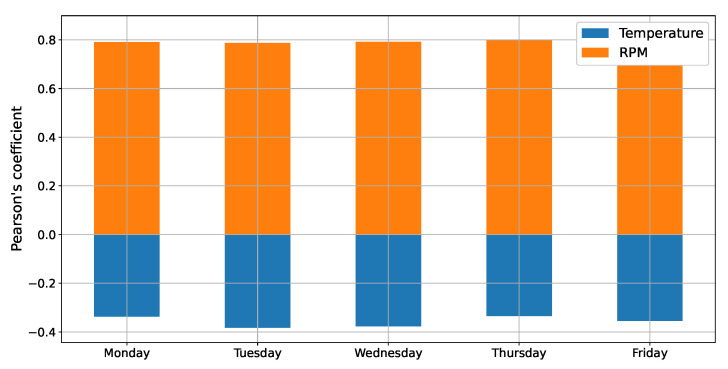
Pearson’s coefficient for each dataset.

To further study the vehicle data, we reanalyzed Equation (9) in which it is possible to see that the mass of CO_2_ expelled is directly proportional to the engine RPM and inversely proportional to the Intake Absolute Temperature (IAT). These correlations are indicated in Figure 6, which shows the Pearson coefficient, considering the instantaneous values of CO_2_ and the values of RPM and Temperature. Pearson’s coefficient for RPM was close to 0.8, meaning that the correlation is positive, i.e., the variables are directly correlated. For the temperature data, the Pearson’s coefficient is less than zero, confirming what was predicted in theory.

### 5.4. Applying TEDA

After calculating CO_2_ emissions, the TEDA algorithm was applied on the instantaneous values related to the amount of gas produced by the vehicle. The only parameter of the algorithm is the threshold (*m*), which defines the sensitivity of the technique as previously described. The higher the value of *m*, the less sensitive the TEDA will be to detect anomalous values (the outliers). Following the same logic, the more the value of *m* decreases, the sensitivity of the algorithm increases. Therefore, it will be more sensitive to variations, detecting a greater number of anomalous values.

It is important to observe that the defined acquisition frequency was equal to 1 Hz, i.e., for every 1 s the data were collected, the mass of CO_2_ value was calculated and this new value served as an input for TEDA. The number of samples for the five datasets is around 1100, totaling trips lasting less than 20 min. The outlier detection algorithm was applied to the datasets, varying the following threshold value: 1.5, 2.0, and 2.5. For each value, the Table 5 presents the total of inliers and outliers, being possible to confirm that as the threshold increases, the number of anomalous values decreases.

**Figure 7:**
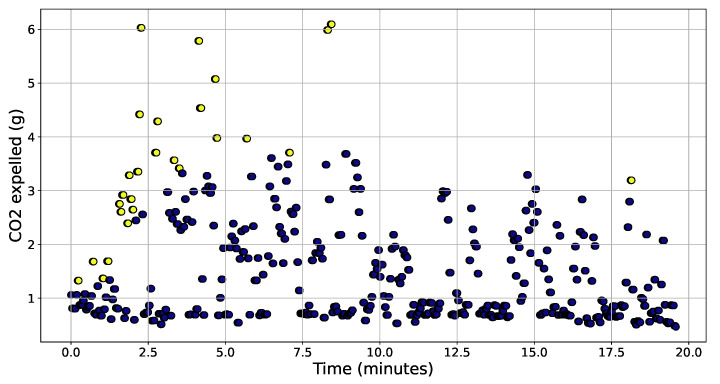
Application of the TEDA algorithm on the Monday dataset using m=1.5.

The CO_2_ values calculated on Monday are shown in Figure 7 in which the outliers are marked in yellow, considering a threshold (*m*) equal to 1.5. The anomalous values were more concentrated at the beginning of the trip, presenting a greater magnitude than most.

**Figure 8:**
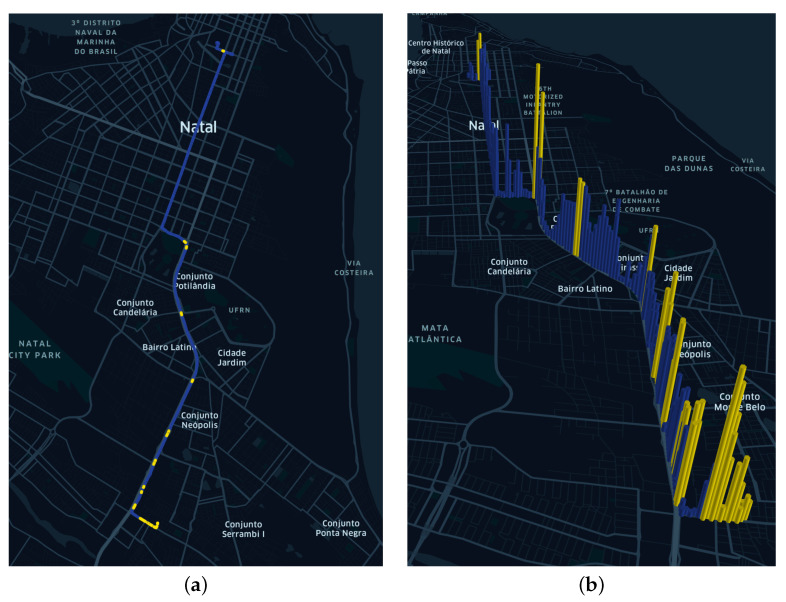
Application of the TEDA algorithm on the Monday dataset using m=1.5. (**a**) Geographical arrangement of outliers (yellow marker). (**b**) Magnitude of CO_2_ values along the route.

The outliers were arranged on the maps presented in Figure 8a, showing the places where they occurred during the total path. Figure 8b shows the height of the bars proportional to the value of CO_2_ emitted.

After all these analyses, we can conclude that the implemented solutions performed efficiently and as expected, presenting valuable information about CO_2_ emissions. The experiments were successfully executed, with the Freematics ONE+ unit and the developed algorithms operating correctly. Finally, the retrieved and processed data were in accordance with expected data in the automobile industry, reinforcing its accuracy for large-scale executions.

An important final remark is about the connectivity of the implemented soft sensor. For the performed experiments, the computed data were stored into a microSD card, which is later processed in a regular computer. However, since the idea is to allow distributed measurements from multiple vehicles, the soft sensors might need to be connected somehow. Actually, the Freematics ONE+ board already supports 4G and Bluetooth protocols, which could support interconnections among multiple units or from one soft sensor to a central server. However, since the proposed TinyML-based soft-sensor approach is executed locally, without requiring any interaction with the Cloud in a different way from other works in the literature, poor connectivity and networking failures are not major concerns for our solution. Nevertheless, when implemented at a larger scale, networking dependability issues should be proper considered, as will be discussed in future works.

## 6. Conclusions

The stringent challenges related to the greenhouse effect and the resulted climatic changes have fostered the development of monitoring and assessment solutions to support the identification of major pollution sources. Among such solutions, the adoption of embedded algorithms to be attached onto vehicles is a promising approach, which may significantly benefit smart cities macro-systems. When smart vehicles become interconnected, cities may better perceive how air pollution is being generated and how public measures may be adopted to relieve its negative impacts.

This work proposed an innovative solution to compute CO_2_ emissions by vehicles directly reading their OBD-II interface. Running on a Freematics ONE+ hardware unit, the proposed algorithms directly gather data provided by the vehicle through the built-in OBD-II scanner in the unit, computing the CO_2_ emissions every 1 s. Until this point, the proposed solution is very useful for many practical scenarios. In addition, this work also proposed a TinyML mechanism to improve the accuracy of the achieved results, eliminating outliers within the produced data stream. Altogether, the proposed approach brought an important contribution to this area, opening a new development trend based on embedded machine learning algorithms in resource-constrained (and cheap) hardware platforms.

The experimental results were very promising, indicating a practical utilization of the implemented solutions. As future works, the deployment of the solution on a group of vehicles is intended, which will transmit computed CO_2_ emissions to the Cloud through 4G or Bluetooth (with support of a smartphone) networking capabilitues available on the Freematics ONE+ unit. By doing so, large-scale monitoring in a city is planned, which may bring additional important results. These next research steps can even be integrated with parallel monitoring approaches to provide a broader perspective about the air quality in a city.

## Figures and Tables

**Table 1:** Summary of recent related works.

Work	OBD-II Reader	Edge Computing	TinyML	Observed Sensors
This work	Freematics ONE+	Yes	Yes	MAP, MAF, IAT, RPM
[28]	ELM-327 *	Mobile App	No	MAP, Speed
[29]	ELM-327 *	No Offline computing	No	MAP, MAF, IAT, RPM
[30]	ELM-327 *	No Offline computing	No	MAP, Speed, AP
[31]	Freematics ONE+	No Offline computing	No	MAP, Speed, RPM, MAF, IAT
[32]	OBD-II reader not specified + Raspberry Pi	No Offline computing	No	MAP, Speed, RPM, MAF, IAT

* ELM-327 has an ATMega644 microcontroller internally, but it is not used for computing in the cited works.

**Table 2:** PIDs related to CO_2_ emissions.

CO_2_ Related PIDs	Unit
MAP (Manifold Absolute Pressure)	kPa
MAF (Mass Air Flow)	g/s
IAT (Intake Absolute Temperature)	K
RPM (Revolutions Per Minute)	RPM

**Table 3:** Fuel conversion constants.

Fuel	Density (g/L)	AFR	${\mathrm{CO}}_{2\mathrm{PL}}$ (g/L)
Gasoline	737	14.7:1	2310
Diesel	850	14.6:1	2660
Ethanol	789	9.0:1	1510

**Table 4:** Data collected from sensors by the Freematics ONE+ unit.

PID	Abbreviature	Unit
Latitude	Lat	° (degrees)
Longitude	Long	° (degrees)
Speed (OBD-II)	Speed	km/h
RPM	RPM	-
Intake Air Temperature	IAT	K
Mass Air Flow	MAF	g/s

**Table 5:** Application of the TEDA algorithm by varying the threshold (*m*): 1.5, 2.0, and 2.5.

Threshold (*m*)	1.5		2.0		2.5	
**Day**	**Inlier**	**Outlier**	**Inlier**	**Outlier**	**Inlier**	**Outlier**
Monday	1087	88	1118	57	1138	37
Tuesday	1002	110	1051	61	1083	29
Wednesday	981	107	1018	70	1055	33
Thursday	1069	94	1128	35	1145	18
Friday	1030	113	1077	66	1094	49

## Data Availability

Not applicable.
